# Improving community well-being through collaborative initiatives at a medical library

**DOI:** 10.5195/jmla.2019.486

**Published:** 2019-07-01

**Authors:** Melissa C. Funaro, Rahil Rojiani, Melanie J. Norton

**Affiliations:** Harvey Cushing/John Hay Whitney Medical Library, Yale University, New Haven, CT, melissa.funaro@yale.edu; School of Medicine, Yale University, New Haven, CT, rahil.rojiani@yale.edu; Harvey Cushing/John Hay Whitney Medical Library, Yale University, New Haven, CT, melanie.norton@yale.edu

## Abstract

**Background:**

In an increasingly digital age, the role of the library is changing to better serve its community. The authors’ library serves health care professionals who experience high levels of stress due to everyday demands of work or study, which can have negative impacts on physical and mental health. Our library is committed to serving the needs of our community by identifying opportunities to improve their well-being.

**Case Presentation:**

Librarians at the Harvey Cushing/John Hay Whitney Medical Library at Yale University developed a group mindfulness program and a space for self-defined personal care to assist health care professionals in alleviating stress. Surveys were used to evaluate the mindfulness program and self-care space.

**Conclusions:**

We successfully implemented two collaborative wellness and self-care initiatives with students and other stakeholders, as demonstrated by program attendance, diverse space use, and positive survey responses for both initiatives. While these endeavors do not replace the need to challenge structural problems at the root of stress in the health care professions, this case report offers a blueprint for other medical libraries to support the well-being of their communities.

## BACKGROUND

As the world becomes increasingly digital, the role of the library is rapidly changing. In a recent article, Adamowski, the 2015 president of the Illinois Library Association and Wheaton Public Library director, is quoted as saying, “We have to always be at the front lines of the needs of the community…Each library was the same a long time ago, but each community is different…[and needs] different services” [1]. Considering this, public and academic libraries have been expanding their functions and evolving the use of their spaces to meet community needs [2–4].

The stress of modern health care professions is well established. The rate of physician burnout is estimated to be around 45%–50% across specialties [5], with similar levels among nurses [6] and emergency department physician assistants [7]. Furthermore, physicians have some of the highest suicide rates, ranging from 1.4 to 2.3 times greater than that of the general population [8, 9]. Nurses also have a suicide risk 1.6 times greater than that of the general population [10].

Health care students can also suffer from debilitating stress. Several studies demonstrate higher levels of depression, stress, anxiety, and suicidal ideation in medical students compared to the general population [11–13]. Between their fourth year of medical school and first year of residency training, 73% of doctors-in-training will meet criteria for psychiatric morbidity, particularly depression, anxiety, or substance abuse [14]. These individuals often do not report symptoms due to fear of stigma and marginalization [15].

Providing wellness activities such as mindfulness meditation or spaces for self-defined care can help health care professionals cope with stress. Mindfulness meditation is the practice of intentionally and nonjudgmentally directing one’s attention to present moment experiences [16]. In addition to improving psychological capacities like attentional and emotional self-regulation [17], mindfulness training has been recognized as a viable treatment to prevent recurrent depression and depressive relapse [18–21] and even as a potential preventative for such disorders, helping to counter behaviors such as rumination that precede and co-occur with depression [22, 23]. Mindfulness meditation in health care professional settings can decrease burnout and psychological stress, improve empathy, and increase well-being among nurses and primary care physicians [24–26], as well as among nursing and medical students [27–29].

Academic libraries are increasingly offering mindfulness meditation sessions [30, 31] and dedicated self-defined care spaces [32–34]. Although no single type of programming will appeal to all individuals, each person has the capacity to identify their own self-care needs, whether a quick nap, meditation, journaling opportunity, or yoga. Providing spaces for wellness activities can also support the diverse spiritual and religious needs of health care professionals and patients, with one study suggesting that spirituality has powerful protective benefits against burnout in the health care field [35]. In fact, creating spaces for prayer is not a new idea as chapels have historically been available at public institutions such as hospitals and prisons and are appearing in airports and shopping malls. Today, these spaces are being rebranded as sacred spaces to be more inclusive of a variety of religious practices [36].

## CASE PRESENTATION

Librarians at the Harvey Cushing/John Hay Whitney Medical Library (CWML) wanted to develop concrete ways to help ease stress and improve the well-being of our health care professional community through providing mindfulness activities. CWML’s community consists of faculty, students, residents, fellows, researchers, nurses, post-docs, and staff from the Yale Schools of Medicine and Nursing, and the adjacent Yale-New Haven Hospital. Librarians approached Being Well at Yale staff for advice on how to start a mindfulness program, and, through informal conversations, they were connected to two students from the School of Medicine and School of Public Health who were interested in collaborating in these efforts. Multiple informal discussions resulted in two concrete initiatives: a group mindfulness program and a wellness/self-care space.

### “Mindfulness on the Medical Campus”

A multifaceted “Mindfulness on the Medical Campus” (MoMC) initiative was launched in the fall of 2015. This initiative consisted of weekly group mindfulness sessions and public events. The first public event, a kick-off for the initiative, was attended by thirty-five students from all health profession schools (nursing, physician associate, medicine, and public health), with most attendees coming from the School of Medicine. The event was advertised through social media (i.e., a Facebook event) as well as CWML, Being Well at Yale, and student wellness websites and email discussion lists. This introductory workshop was led by Rahil Rojiani, the collaborating student from the Yale School of Medicine. This event interwove guided meditations, a lecture on the neuroscience and cognitive psychology of meditation, and a question-and-answer period focusing on how mindfulness meditation is useful for relieving stress and building resilience.

The MoMC weekly program consisted of 30-minute mindfulness sessions held at 4:30 p.m. once or twice a week (Tuesdays and/or Thursdays). The sessions took place in the library and were facilitated by the 2 collaborating students, given their meditation backgrounds. The meditation sessions were promoted through blog posts on the CWML website, events on the Yale School of Medicine’s calendar, and flyers posted throughout the library and in various key locations around the medical campus, as well as emails disseminated via an email discussion list. This email list was generated after the kick-off event and has steadily grown to over 200 subscribers. Attendees are asked to sign up at the sessions and through links on the blog posts, emails, and flyers.

The first year of mindfulness sessions ran from September 2015 to May 2016, coinciding with the health profession schools’ schedules, with a total of 36 sessions. Participants sat in chairs arranged in a circle to accommodate all bodies (ranging in size and flexibility) and experience levels. Sessions consisted of 5–10 minutes of brief introduction to meditation for beginners, followed by 15–20 minutes of guided meditation and 5 minutes of sharing, reflections, and questions. Attendance for this first year averaged 9.35 (standard deviation [SD]=4.02) participants per session.

In collaboration with Integrative Medicine at Yale and with the help of a Student Wellness Grant from the Wellness Project and the office of the secretary and vice president for student life, the first year of the MoMC concluded with a lecture by Judson Brewer, an addiction psychiatrist with more than 20 years of combined personal mindfulness training and neuroscience research on mindfulness. His lecture, “Cultivating Resilience in the Face of Burnout,” was held at 1:00 p.m. on April 8, 2016. The event was designed for attendees to learn about stress, resilience, and the neuroscience behind mindfulness. The event was publicized through various channels: the CWML website, flyers posted throughout campus, Facebook, and email discussion lists. An event invitation was created in Eventbrite for people to register in order to estimate attendance for logistical planning. Over 120 individuals attended the event, including faculty, staff, and students from the health professional schools at Yale University as well as people from the larger New Haven community. Integrative Medicine at Yale helped with advertising as well as funding to cover the cost of refreshments. The Student Wellness Grant covered the cost of travel and the honorarium for Brewer. The CWML provided the space for the event.

With the first year of the MoMC program ending and students who led the program graduating, the program was in jeopardy. To gauge interest in continuing the program, a survey was sent in August 2016 to the MoMC email discussion list for evaluation and feedback. Of the 29 respondents, 84% said the meditation sessions were helpful, 16% said they were moderately helpful, and none said they were unhelpful. There was variability in the number of sessions that respondents attended: 31% of respondents attended 1–3 sessions, 16% attended 4–6 sessions, 16% attended 7–9 sessions, and 37% attended 10 or more sessions. Reasons for attending the sessions also varied, with the most common being to reduce stress or anxiety, to improve general well-being, and to develop a more positive outlook on life.

Ten survey respondents left comments. Consistent with a grounded theory approach [37], two themes emerged from the qualitative data. The first theme was the difficulty of session timing. Five people commented that the time of the session was difficult because they had to pick up their children. Some respondents commented that having children in daycare and other evening obligations prevented them from attending evening sessions, but that they would attend if sessions were held earlier in the day.

The second theme was the desire for the program to continue or expand (e.g., more times or more speakers). Comments highlighting this theme also shared positive feedback:

This is a great program! I was happy to have this as a way to meditate with my friends and colleagues and to learn the practice. It helped to get me started and has been a positive influence in my life.

I have found this Mindfulness @Yale program in the Med Library to be a rewarding experience, which has taught me a lot about meditation and tools for dealing [with] the daily work-related stress. It has been well organized and the instruction has been really been *[sic]* world-class. Thank you for making this Mindfulness @Yale program available; I really hope to see more of this type of program at the University.

I sincerely hope that the mindfulness program will continue, possibly even expand, and that the number of participants will increase as the appreciation of the incredible benefits of mindfulness spreads around.

Librarians chose to find a way to continue the program, making a few changes for the following year. Collaborating with skilled students to lead mindfulness sessions was beneficial, but this benefit was unfortunately transient due to graduations and other school obligations. Thus, the next school year began with weekly and eventually twice-weekly 30-minute sessions of guided meditation led by experienced, professional meditation instructors from Being Well at Yale. This shift was crucial to institutionalizing the program and ensuring a more stable, long-term commitment to the sessions. The sessions were moved to begin at 12:15 p.m. to accommodate the needs of Being Well at Yale staff and participants with childcare responsibilities. These sessions started in September 2016 and are continuing to this day. From September 2016 to May 2017, attendance averaged 6.30 (SD=2.28) participants per session.

### Wellness and self-care space

We felt that mindfulness programming alone was not enough, particularly given the time and scheduling constraints that many health care professionals experienced and the varying needs of different individuals. Therefore, we sought to facilitate personally defined wellness through providing an accessible, dedicated, drop-in self-care space for individuals to engage in these activities and further support students with a variety of religious or spiritual orientations.

One of the 2 initial collaborating students, Rahil Rojiani, applied for and obtained a small competitive grant of $1,000 from the Student Wellness Project. After receiving this funding, we reached out to the health care professional schools via email to form a small committee to facilitate the creation of a space dedicated to wellness and self-care. The resulting committee consisted of 2 medical students and a public health student, who identified a small study room in the CWML and, with support from the CWML director, transformed the room into a nondenominational “Wellness and Self-Care Space.” The funding from the grant was used to purchase a yoga mat, meditation cushions, prayer rugs, a single journal that was left in the room for personal reflection, a whiteboard for community expression and inspiration, and art and décor with a soothing meditative theme (e.g., a student-created sign, a nature painting, artificial plants). The small room was ideal for one person to do yoga and could comfortably fit two people for seated wellness activities.

**Figure 1:**
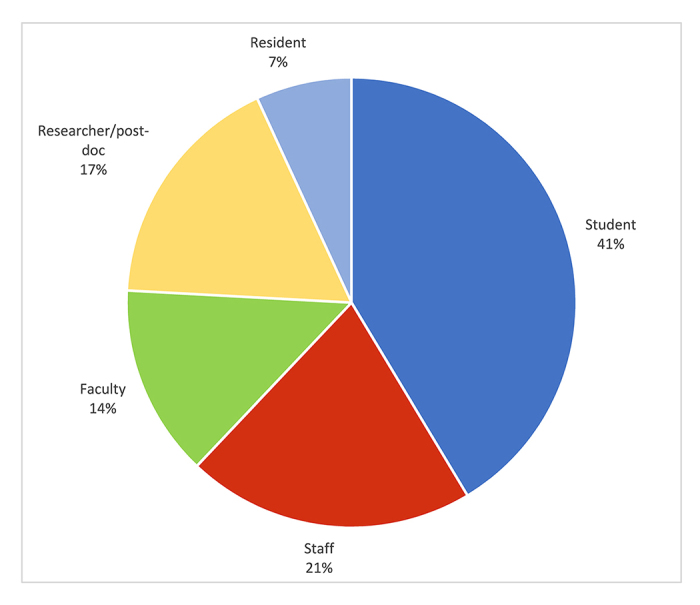
Who uses the wellness and self-care space

This space has been well-received. A 4-question paper survey (multiple choice and free text) was placed in the room at various well-spaced times between September 2016 and July 2017. Twenty-nine individuals provided feedback. Students were the most frequent users (41%), but staff (21%), researchers/post-doctoral fellows (17%), faculty members (14%), and resident physicians (7%) also used the space (Figure 1).

Thirteen of the survey respondents provided comments. Consistent with a grounded theory approach [37], 3 themes emerged. The first was feedback for improvement: the most common suggestion was for a better way to indicate that the room was occupied. The second theme was general appreciation of and gratitude for the space (e.g., “This is a tremendous space…Thank you!,” “I love this space,” “More rooms please…very nice”). The third theme was that the space met a previous unfilled need for a safe, private area for prayer:

It’s awesome. I would pray in the stacks. It was awkward. This is amazing, so helpful, peaceful.

Thank you!!

Thank you for this room. Makes my study process easier. I can pray here and save time because before I needed to go to the chapel. It takes more time.

**Figure 2:**
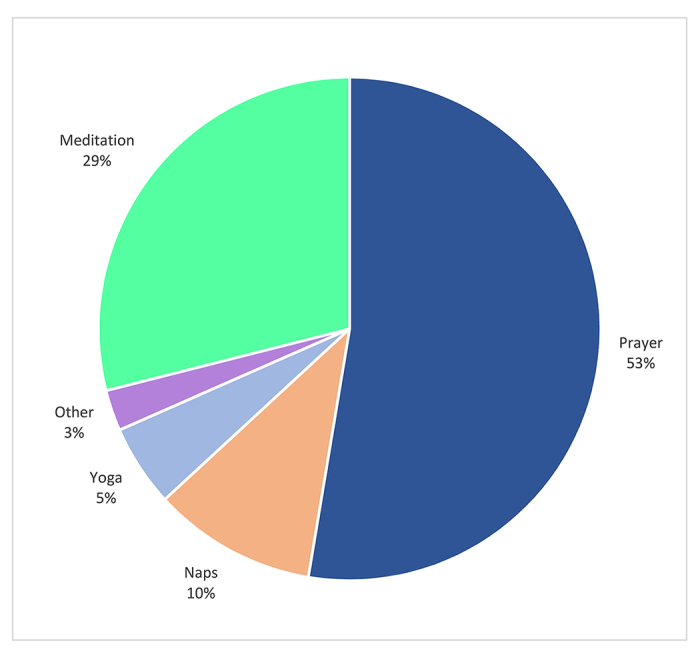
Use of the wellness and self-care space

These survey responses align with the quantitative data from the survey indicating how the room was typically used: primarily for prayer (53%) and meditation (29%) but also for naps (10%), yoga (5%), or other purposes (3%) (Figure 2).

A couple of key lessons were learned through this process. First, community collaboration—particularly with students—is crucial for successful execution and community investment. For both initiatives, this included involving students in planning, working with larger student and faculty organizations to hold events, and teaming with larger Yale University wellness initiatives for funding, promotion, and continued mindfulness facilitation. The CWML could not have successfully accomplished these initiatives alone and certainly not without the broad reach it had. Further, involvement of multiple groups led to more people feeling invested in participating in and promoting these initiatives.

Another lesson is the importance of a feedback-driven, iterative development process. In the two years of the MoMC, several changes were made to meet the needs of our community. For example, survey responses helped identify timing conflicts for those with children, resulting in time changes, and difficulties with communicating self-care room occupancy, resulting in better “in use” signage. We also iteratively improved the program and space through involving a student committee with varying backgrounds and interests. It was through this process that we identified the ongoing struggle of Muslim health care students to find a safe private prayer space on campus, resulting in our inclusion of a prayer rug.

We hope to continue employing this process of feedback to improve the program. For example, we are unsure of the reasons that attendance dropped in the second year after the timing change. Our theories include settling of an initial hype in the first year, a kickoff event only for the first year, timing difficulties for students in class, and changes in facilitators. Future surveys may help us elicit this information and identify timing and facilitation styles that suits the needs of all our community members.

## DISCUSSION

In this increasingly digital age, some academic libraries are evolving their services through introducing wellness programs such as mindfulness meditation and self-care spaces [30–34]. These programs share the desire to meet the wellness needs of their unique communities, whether it is through mindfulness meditation sessions or self-defined care spaces. Our case study differs from examples we found because we reported on quantitative and qualitative methods through surveys and attendance data to provide examples of how our program is being used.

It is more important than ever in our current climate that health care professionals have an opportunity to mitigate the stress that is unfortunately so inherent in the profession. The library can offer space and staff time to create and promote self-care and wellness opportunities to help mitigate some of the stresses of being a health care student or professional. The CWML is particularly well suited to this task given its central, accessible location with the high foot-traffic of the health care community, as opposed to other collaborators based on the main Yale College campus or collaborating groups without an established physical space. Our MoMC initiative was developed to meet the needs of our community and will differ from wellness initiatives at other institutions, because each community is unique in both its resources and needs.

A few important observations may be helpful for other libraries hoping to replicate these initiatives. First, given the current national political environment, including growing Islamophobia [38] and broader anti-religious sentiment on university campuses [39], the library has the capacity to provide a safe space for all community members. Second, given the advantage of collaborative efforts, these endeavors have great benefit with minimal financial costs. Third, group activities outside of the high-stress context of work or classes provide a wonderful opportunity for community building and even the potential for collaboration.

Finally, we must convey that while the steps that we took provided an opportunity to mitigate stress and improve well-being, such steps were aimed at individual rather than structural methods of accomplishing this change. Such individual-level initiatives must also be met by structural changes to improve the working and studying conditions of our community. Unless we also address these systemic problems, we fail to uncover and solve the deeper causes of distress and burnout.

Our case study provides a detailed map of our own journey toward improved wellness initiatives in a medical library through regular, accessible mindfulness sessions and a personal self-care space. We hope this case study provides other institutions with the motivation and details to implement similar wellness initiatives. We believe our case report and survey responses show a need for these services and how the library can be a natural collaborator in providing these types of services and spaces. This is further evidenced by the fact that these types of spaces and programs are increasingly being introduced in libraries and other types of public spaces.
